# Effects of a Web-Based Lifestyle Intervention on Weight Loss and Cardiometabolic Risk Factors in Adults With Overweight and Obesity: Randomized Controlled Clinical Trial

**DOI:** 10.2196/43426

**Published:** 2023-06-27

**Authors:** Jan Kohl, Judith Brame, Christoph Centner, Ramona Wurst, Reinhard Fuchs, Matthias Sehlbrede, Iris Tinsel, Phillip Maiwald, Urs Alexander Fichtner, Christoph Armbruster, Erik Farin-Glattacker, Albert Gollhofer, Daniel König

**Affiliations:** 1 Department of Sport and Sport Science University of Freiburg Freiburg Germany; 2 Section of Health Care Research and Rehabilitation Research (SEVERA), Medical Center Faculty of Medicine University of Freiburg Freiburg Germany; 3 Department of Sport Science Institute for Nutrition, Exercise and Health University of Vienna Vienna Austria; 4 Department of Nutritional Sciences Institute for Nutrition, Exercise and Health University of Vienna Vienna Austria

**Keywords:** web-based intervention, randomized controlled trial, dietary energy density, weight loss, obesity, overweight, cardiometabolic risk factors

## Abstract

**Background:**

The high proportion of people with overweight and obesity has become a worldwide problem in recent decades, mainly due to health consequences, such as cardiovascular diseases, neoplasia, and type 2 diabetes mellitus. Regarding effective countermeasures, the digitization of health services offers numerous potentials, which, however, have not yet been sufficiently evaluated. Web-based health programs are becoming increasingly interactive and can provide individuals with effective long-term weight management support.

**Objective:**

The purpose of this randomized controlled clinical trial was to evaluate the effectiveness of an interactive web-based weight loss program on anthropometric, cardiometabolic, and behavioral variables and to compare it with a noninteractive web-based weight loss program.

**Methods:**

The randomized controlled trial included people who were aged between 18 and 65 years (mean 48.92, SD 11.17 years) and had a BMI of 27.5 to 34.9 kg/m^2^ (mean 30.71, SD 2.13 kg/m^2^). Participants (n=153) were assigned to either (1) an interactive and fully automated web-based health program (intervention) or (2) a noninteractive web-based health program (control). The intervention program focused on dietary energy density and allowed for dietary documentation with appropriate feedback on energy density and nutrients. The control group only received information on weight loss and energy density, but the website did not contain interactive content. Examinations were performed at baseline (t0), at the end of the 12-week intervention (t1), and at 6 months (t2) and 12 months (t3) thereafter. The primary outcome was body weight. The secondary outcomes were cardiometabolic variables as well as dietary and physical activity behaviors. Robust linear mixed models were used to evaluate the primary and secondary outcomes.

**Results:**

The intervention group showed significant improvements in anthropometric variables, such as body weight (*P*=.004), waist circumference (*P*=.002), and fat mass (*P*=.02), compared with the control group over the course of the study. The mean weight loss after the 12-month follow-up was 4.18 kg (4.7%) in the intervention group versus 1.29 kg (1.5%) in the control group compared with the initial weight. The results of the nutritional analysis showed that the energy density concept was significantly better implemented in the intervention group. Significant differences in cardiometabolic variables were not detected between the 2 groups.

**Conclusions:**

The interactive web-based health program was effective in reducing body weight and improving body composition in adults with overweight and obesity. However, these improvements were not associated with relevant changes in cardiometabolic variables, although it should be noted that the study population was predominantly metabolically healthy.

**Trial Registration:**

German Clinical Trials Register DRKS00020249; https://drks.de/search/en/trial/DRKS00020249

**International Registered Report Identifier (IRRID):**

RR2-10.3390/ijerph19031393

## Introduction

The past decades have been characterized by a sharp increase in BMI [1-4]. In 2016, 39% of adults worldwide were overweight, with the prevalence in countries, such as Germany, being significantly higher at over 50% [5]. The increase in overweight and obesity is a cause of concern, as a high BMI is associated with health consequences, such as cardiovascular diseases, neoplasia, and type 2 diabetes mellitus, which can lead to impaired quality of life, premature frailty, and increased mortality [6,7]. In addition to the individual health consequences for a significant proportion of the world’s population, the financial costs to society are substantial. Direct costs arise from the treatment of obesity and the associated comorbidities, and indirect costs arise from, for example, work absences due to illness or early retirement [8,9]. It was demonstrated that the median cost of health care (direct costs) was 12% higher for people with overweight and 36% higher for people with obesity than for people with a normal weight [10].

Although there are specific and evidence-based dietary and physical activity recommendations to prevent and treat obesity and its associated comorbidities, these behaviors often cannot be implemented and established in the long term. Systemic and environmental drivers have a lasting effect on behavior and make long-term behavior change difficult [1]. For example, the food environment affects the control of food intake, whereby constantly available and energy-dense foods could lead to an increase in energy intake. These external signals influence the neural regulation of energy balance unconsciously, and a mere recommendation to reduce food intake is thus ineffective for long-term weight loss [11]. In this context, emerging evidence supports the relevance of dietary energy density in modifying energy intake and suggests it is a useful approach to weight loss [12-16].

In addition to potential solutions from nutrition or sports science, ways to improve preventive and therapeutic lifestyle interventions to counter the global trend of rising BMI and associated comorbidities are also being sought at the level of the delivery medium used. It is now well established that face-to-face lifestyle interventions can be effective in preventing and treating obesity and its associated diseases [17-19]. As digitization and technological capabilities continue to advance, web-based interventions to promote physical activity and healthy eating have increasingly come into focus to reduce body weight [20,21]. With potentially unlimited distribution, practically no waiting times, and low barriers, web-based interventions are intended to provide effective and low-cost care over the usual period of a face-to-face lifestyle intervention [22,23]. In terms of effectiveness and cost, there is preliminary evidence that web-based interventions can be used reasonably to treat overweight and obesity, as well as cardiovascular risk factors [24-28].

Despite promising approaches, the effectiveness of web-based interventions is still under debate. In particular, longer-term effects and interactive web-based interventions require further investigation [21]. In addition, it should be noted that web-based interventions can be designed and structured very differently and that these differences can also influence the results to a large extent. In this context, it seems that interactive and tailored programs have greater effects on weight loss than informative websites [21,26,29].

Using interactive web-based interventions and addressing dietary energy density are considered 2 potential approaches for successful weight management, but to the best of our knowledge, they have not been studied in combination. The purpose of this randomized controlled clinical trial was to investigate the effectiveness of an interactive and fully automated web-based weight loss program focusing on dietary energy density in adults with overweight and obesity. Therefore, the study compared interactive and noninteractive web-based interventions, as both interventions have an unlimited reach and availability due to the lack of human involvement. We hypothesized that the interactive program would result in statistically significant improvements with small to medium effect sizes. Moreover, we assumed that these improvements would be significantly more pronounced in the interactive program than in the noninteractive program. The interactive weight loss program is part of a multimodal health program developed by a German health insurance company. This multimodal health program addresses individual health goals, such as weight loss, physical fitness, healthy eating, and smoking cessation. The different modules provide an individually tailored health intervention depending on the health goal and status. Each of the modules enables interactive health intervention for the prevention of noncommunicable diseases via selectable activities and corresponding feedback. This clinical substudy is part of an evaluation of a German-language web-based lifestyle intervention coordinated by the Section for Health Services Research and Rehabilitation Research (SEVERA) at the University Medical Center Freiburg [30,31]. The web-based weight loss program was investigated both in an online questionnaire study [32] and regionally in southwest Germany with medical variables in this clinical substudy.

## Methods

### Study Protocol

The main methodological points of this randomized controlled clinical trial with relevance to this paper are described below. A detailed description of the study methods can be found in the study protocol, which has already been published [30]. Additional information on the related online questionnaire study can be found elsewhere [31].

### Study Design

Study participants in the online questionnaire study “weight loss” who were living in southwest Germany (postcode area: 79) were additionally invited to the Department of Sport and Sport Science of the University of Freiburg for the clinical substudy [30,31]. While changes in body weight were also reported by the participants in the online questionnaire study, in this subgroup, in which participants appeared in person for examination, additional clinical parameters and laboratory variables were collected. Medical examinations took place at baseline (t0), after the 12-week intervention (t1), and after an additional 6 months (t2) and 12 months (t3) of follow-up. During these medical examinations, participants had the opportunity to provide qualitative feedback. Besides these medical examinations, the participants of the clinical trial received the same web-based intervention as the participants of the online questionnaire study.

The study included intervention and control groups with automated randomization after completing the questionnaire at t0. Permuted block randomization was performed to obtain an approximately equal distribution in both study groups. Variable block sizes of 4, 6, and 8 were used for this purpose. The allocation sequence was created by SEVERA using RITA software (Version 1.50; Universität zu Lübeck) [30,31]. Randomization was performed automatically after online registration of the study participants. Because study participants can recognize their allocated intervention, blinding was not possible. Outcome assessors were blinded until completion of the analysis [30].

### Participants

Participants aged 18 to 65 years in the online questionnaire study and residing in southwest Germany were additionally eligible to participate in the clinical substudy. The inclusion criteria for the clinical trial were a BMI of 27.5 to 34.9 kg/m^2^ and no pregnancy or breastfeeding. Furthermore, subjects were required to be in good health, especially without any illnesses where weight reduction could possibly lead to subsequent health problems. In the case of existing health problems and illnesses, a medical certificate had to be submitted confirming eligibility for participation in the study. Since the intervention was purely web-based, appropriate computer skills were required for online registration and participation. The sample size was calculated to be 150 based on the primary outcome of body weight [31].

Recruitment of the participants took place online and offline through various media, such as local newspapers, flyers, and Google advertisements. Through the various recruitment media, interested individuals were directed to an open-access landing page. On the landing page, people were able to find out about the study and register. Written informed consent was obtained for registration. After registration, automatic randomization was performed immediately, and the clinical substudy staff contacted the prospective study participants. During the contact, information about the study was provided again, the inclusion and exclusion criteria were verified, and an appointment was made for the first medical examination. With the successful completion of the first medical examination, the study enrollment was completed. As an incentive, participants in the clinical trial received an activity tracker, which also served as a measurement tool to record physical activity. Specific information on sample calculation and recruitment can be found in the detailed study protocol [30].

### Intervention

Both interventions were fully automated and without human involvement. The intervention group received an interactive web-based health program. The multimodal web-based health program could provide personalized intervention depending on health goals. The web-based health program was frozen for the evaluation to create a consistent study version. For this study, all participants in the intervention group received the health program’s weight loss module. This module of the health program runs for 12 weeks and aims to achieve long-term behavior change. The 12-week intervention was divided into 3 phases. In phase 1, users should try out and get to know the program (weeks 1-3). In phase 2, consolidation of the new behavior should occur (weeks 4-6). In the final phase 3, the new habits should be strengthened (weeks 7-12). Despite this division, the program was freely usable and did not follow a linear sequence. All information texts, videos, and activities could be accessed at any time, allowing the program to be used according to individual pace or need.

The focus of the interactive weight loss program was on reducing dietary energy density. For this, the program offered the possibility to log the diet and receive feedback accordingly in terms of energy density, energy intake, and macronutrients. The primary goal was not to have the participants lose as many kilograms as possible in terms of a crash diet. Rather, the goal was to achieve relatively slow weight loss, thereby primarily losing fat mass and maintaining the resting metabolic rate [33]. Therefore, the participants had the choice whether they wanted to lose 3 or 5 kg during the 12-week intervention. However, a higher weight loss was not prevented by the program. Moreover, the health program could be further individualized with numerous selectable activities. For the “weight loss” health goal, these included activities such as achieving 2 servings of fruit and 3 servings of vegetables, drinking at least 1.5 liters of water per day, or reaching 10,000 steps per day. The interactivity of the weight loss program was generated via the selectable activities and logging of the diet with corresponding visualized feedback.

In addition to this interactive content, there was an extensive knowledge area. This knowledge area included evidence-based articles on dietary energy density, healthy eating, and weight loss. Some of these articles were part of a weekly task and were staggered throughout the 12-week intervention. Besides the evidence-based information, the intervention offered a comprehensive collection of recipes to support users in practical application.

In contrast, the control group received noninteractive web-based information on how to lose weight by lowering dietary energy density while eating healthy. This noninteractive information was transmitted by short articles and was intended to serve the transfer of knowledge. This was a static intervention, meaning that no change in content occurred over the course of the 12 weeks.

Both groups were allowed to use or rerun their program following the 12-week intervention period. Thus, the corresponding program was freely available during the follow-up. Further details about the intervention can be found in the study protocol [30,31].

### Outcome Variables

The primary outcome of the study was body weight. This was measured using the validated bioelectrical impedance scale Seca mBCA 515 (Seca GmbH & Co KG) [34-36]. The standardized measurement was performed in underwear, without accessories, such as glasses and jewelry, after 12 hours of fasting, and with an empty bladder.

In addition, behavioral and physiological variables were defined as secondary outcomes. In the behavioral domain, dietary and physical activity behaviors were recorded. Seven-day dietary records were generated at each of the 4 measurement time points using NutriGuide Plus software (Version 4.8; Nutri-Science GmbH). Energy intake, dietary energy density (excluding beverages), and macronutrients were evaluated using the dietary data. Physical activity was assessed using the activity tracker Fitbit Charge 3 (Fitbit, Inc) and the long version of the International Physical Activity Questionnaire (IPAQ-L; German) [37]. A minimum of 5 reliable days was required for each of the 1-week diet (dietary record) and physical activity (activity tracker) data collections to be included in the analysis.

In addition to the primary outcome of body weight, other anthropometric variables were measured or calculated. Thus, body height, BMI, waist circumference, fat mass, and fat-free mass were recorded using the bioelectrical impedance analysis scale Seca mBCA 515, the stadiometer Seca 274, and the measuring tape Seca 201 (Seca GmbH & Co KG). Blood samples were collected, and blood glucose (fasting glucose and glycated hemoglobin [HbA_1c_]) and blood lipids (total cholesterol, high-density lipoprotein [HDL] cholesterol, low-density lipoprotein [LDL] cholesterol, and triglycerides) were analyzed by the Medical Care Center (MVZ) Clotten in Freiburg. Blood pressure was measured using a validated measuring device (Boso Medicus Exclusive, BOSCH + SOHN GmbH & Co KG). A detailed list of all variables collected can be found in the study protocol [30].

### Data Analysis

R (Version 4.1.3; R Core Team) and R Studio (Version 2021.09.1; Posit PBC) were used for statistical analysis and creation of the graphs. Statistical analysis of all variables was performed with robust linear mixed models using the R packages lme4 [38] and robustlmm [39]. The significance level was set at .05 for all comparisons. Graphs of descriptive results of anthropometric variables were created using the R package ggplot2 [40].

Per-protocol (PP) and intention-to-treat (ITT) analyses were performed. In the PP analysis, only subjects without missing values for the respective variable were included (complete cases). If missing data were available for individual outcomes, fewer study participants were considered accordingly. In the ITT analysis, all randomized cases were included. Missing values were imputed by multiple imputation (n=50) using the R package micemd [41]. PP and ITT analyses showed similar results across all variables. Owing to the large number of variables, only the ITT analysis has been presented here.

### Ethical Considerations

This study followed the principles of the Declaration of Helsinki. The study was approved by the Ethics Committee of the University of Freiburg on July 25, 2019 (vote number: 237/19). A clinical pilot study was conducted (vote number: 409/18, DRKS00016512), resulting in minor changes to the study protocol. These changes were positively assessed by the Ethics Committee (date of approval: October 22, 2019; protocol version: amendment 01). Written informed consent was provided by all participants prior to study inclusion.

## Results

### Participants

**Figure 1:**
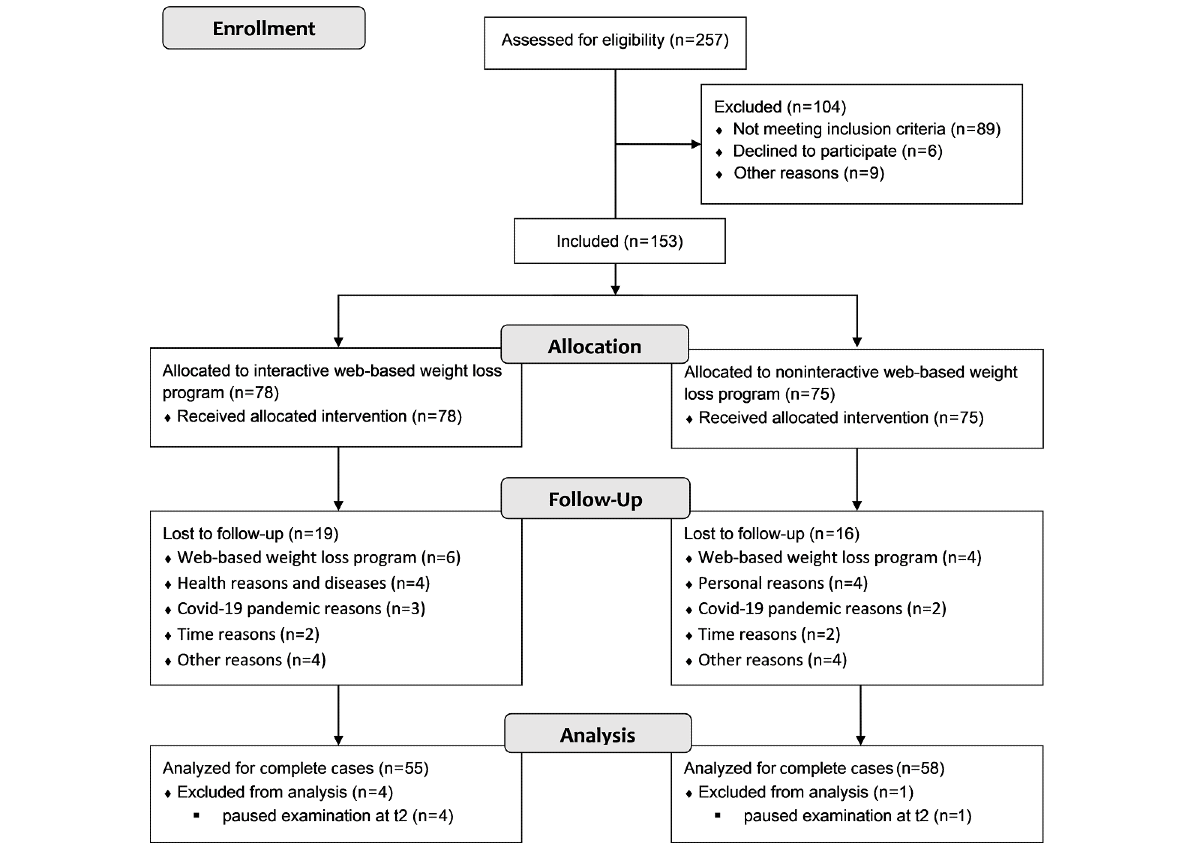
Flow chart depicting participant recruitment and dropout. t2: 6-month follow-up.

Recruitment of participants took place from January 2020 to July 2020. During this period, 257 interested individuals registered for the clinical substudy. After screening (telephone interview and preliminary examination), 153 subjects were included in the study, and they completed the baseline measurement. There were 35 dropouts throughout the study. None of the health reasons of the dropouts were associated with the intervention. Since 4 study subjects did not attend the t2 examination due to the COVID-19 pandemic, they could not be included in the PP analysis (complete cases). The study participant flow is shown in Figure 1. The baseline characteristics of the included participants are summarized in Table 1.

**Table 1:** Baseline (t0) characteristics of the study participants.

Variable		All participants (N=153)	Intervention group (n=78)	Control group (n=75)
Age (years), mean (SD)		48.92 (11.17)	49.12 (11.36)	48.72 (11.05)
**Sex, n (%)**				
	Male	44 (28.8)	20 (25.7)	24 (32.0)
	Female	109 (71.2)	58 (74.3)	51 (68.0)
Body weight (kg), mean (SD)		88.39 (10.65)	88.42 (10.15)	88.36 (11.21)
Body height (m), mean (SD)		1.69 (0.08)	1.69 (0.07)	1.70 (0.08)
BMI (kg/m^2^), mean (SD)		30.71 (2.13)	30.88 (2.20)	30.54 (2.05)
Dropouts, n (%)		35 (22.9)	19 (24.4)	16 (21.3)

### Body Weight and Body Composition

**Figure 2:**
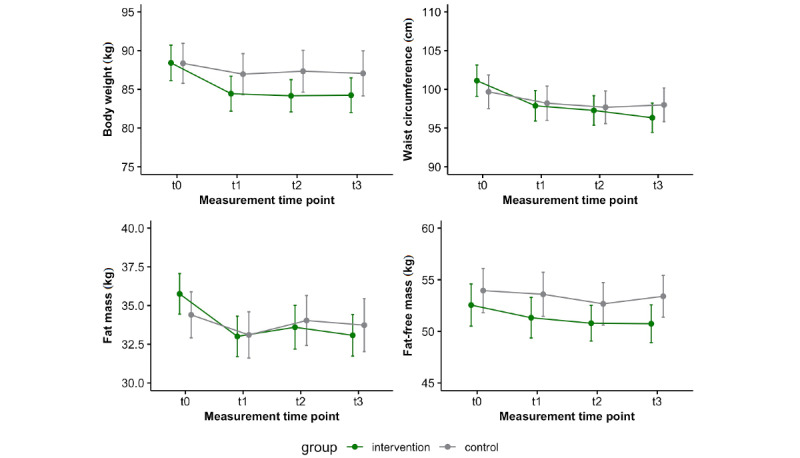
Data (mean and 95% CI) of anthropometric variables in the intervention (n=78) and control (n=75) groups (intention-to-treat analysis). The time points were baseline (t0), after the 12-week intervention (t1), and after an additional 6 months (t2) and 12 months (t3) of follow-up.

Figure 2 shows the descriptive course of the anthropometric variables in the ITT analysis. The participants in the intervention group lost an average of 3.98 kg (4.5%) of their baseline body weight after 3 months (t1) and 4.18 kg (4.7%) after an additional 12 months (t3), whereas the participants in the control group lost only 1.40 kg (1.6%) and 1.29 kg (1.5%), respectively. Participants in the intervention group showed significantly greater body weight and fat mass loss (all *P*<.05). In the long term (t0-t3), a greater decrease in waist circumference was also observed compared to the findings in the control group (Table 2). Effect sizes were small for changes in body weight and fat mass, and small to medium for waist circumference in the intervention group (Multimedia Appendix 1). In the intervention group, fat-free mass decreased significantly at t1 and t3 compared with the findings at t0, with no significant differences compared with the findings in the control group.

**Table 2:** Results of the robust linear mixed model of anthropometric variables (intention-to-treat analysis).

Predictor		Body weight (kg)			Fat mass (kg)			Fat-free mass (kg)			Waist circumference (cm)	
		Estimate^a^	*P* value	Estimate^a^		*P* value	Estimate^a^		*P* value	Estimate^a^		*P* value
Intercept		87.80 (2.62)	<.001	37.27 (1.70)		<.001	49.24 (1.92)		<.001	102.42 (2.44)		<.001
**Time^b^**												
	t0-t1	−5.44 (1.34)	<.001	−3.94 (1.02)		<.001	−1.41 (0.59)		.02	−4.44 (1.28)		<.001
	t0-t2	−6.00 (1.47)	<.001	−3.64 (1.13)		.001	−1.01 (0.72)		.16	−5.26 (1.36)		<.001
	t0-t3	−5.87 (1.39)	<.001	−4.03 (1.09)		<.001	−1.60 (0.63)		.01	−7.21 (1.45)		<.001
Group (control)		−0.22 (1.67)	.89	−1.63 (1.08)		.13	1.49 (1.23)		.22	−1.36 (1.55)		.38
**Time×group (control)**												
	t0-t1	1.91 (0.85)	.03	1.28 (0.65)		.048	0.62 (0.38)		.10	1.34 (0.82)		.10
	t0-t2	2.37 (0.94)	.01	1.43 (0.70)		.04	0.03 (0.43)		.95	1.63 (0.87)		.06
	t0-t3	2.41 (0.84)	.004	1.64 (0.70)		.02	0.69 (0.39)		.08	2.75 (0.87)		.002

^a^Unstandardized regression coefficients with standard errors in parentheses.

^b^The time points were baseline (t0), after the 12-week intervention (t1), and after an additional 6 months (t2) and 12 months (t3) of follow-up.

Compared with the body weight levels at baseline, 42.3% (33/78) of participants in the intervention group and 26.7% (20/75) in the control group had lost more than 5% of their body weight at t3. The results of the PP analysis for the primary outcome of body weight were comparable to those of the ITT analysis. Compared with the findings at baseline, the PP analysis also showed significant time and interaction effects at all measurement time points (data not shown).

### Behavioral Variables

Energy density decreased significantly from baseline (t0) to t1 (*P*=.02) and t3 (*P*=.04) in the intervention group compared with the control group (Table 3; Multimedia Appendix 2), with small to medium effect sizes within the group (Multimedia Appendix 3). The energy intake in the intervention group decreased significantly by approximately 200 kcal/day from t0 to t1 (*P*=.01), with no significant differences between the groups (Multimedia Appendix 2). Except for fat intake, no macronutrient changed significantly. The fat intake in the intervention group decreased significantly from t0 to t1 (*P*<.001) and t3 (*P*=.02); however, only from t0 to t1, the reduction was significantly greater than the reduction in the control group (*P*=.02).

**Table 3:** Descriptive statistics of behavioral variables (intention-to-treat analysis).

Variable and group^a^			t0^b^, mean (SD)		t1^b^, mean (SD)		t2^b^, mean (SD)		t3^b^, mean (SD)
**Energy density (kcal/g)**									
	Intervention	1.63 (0.37)		1.38 (0.30)		1.55 (0.30)		1.48 (0.33)	
	Control	1.64 (0.32)		1.58 (0.32)		1.67 (0.33)		1.63 (0.32)	
**Energy intake (kcal/day)**									
	Intervention	1957.51 (585.16)		1760.09 (519.79)		1912.33 (492.67)		1821.87 (601.90)	
	Control	1999.51 (566.26)		1882.71 (473.25)		1876.10 (483.42)		1910.56 (477.48)	
**Protein intake (g/day)**									
	Intervention	72.38 (21.96)		70.37 (22.27)		72.21 (72.48)		72.48 (24.15)	
	Control	77.01 (20.17)		73.63 (17.59)		72.94 (16.70)		73.59 (17.73)	
**Carbohydrate intake (g/day)**									
	Intervention	209.94 (69.71)		190.65 (64.13)		202.35 (59.00)		193.27 (69.21)	
	Control	209.69 (65.77)		195.65 (67.48)		199.67 (63.69)		199.45 (55.56)	
**Fat intake (g/day)**									
	Intervention	79.93 (28.26)		67.47 (23.13)		76.51 (26.79)		72.44 (27.77)	
	Control	81.43 (27.92)		77.64 (23.73)		76.15 (20.79)		77.98 (23.01)	
**Alcohol intake (g/day)**									
	Intervention	8.50 (10.95)		7.19 (9.10)		5.97 (6.49)		7.29 (6.70)	
	Control	9.32 (12.59)		8.13 (8.89)		7.68 (10.64)		9.11 (10.44)	
**Fiber intake (g/day)**									
	Intervention	21.23 (7.78)		21.36 (8.67)		22.50 (7.71)		22.62 (9.37)	
	Control	21.27 (14.26)		19.57 (7.67)		20.98 (7.21)		20.52 (6.38)	
**Physical activity (Fitbit; min/week)**									
	Intervention	2231.85 (512.28)		2157. 07 (709.80)		2193.20 (537.33)		2143.04 (497.93)	
	Control	2191.72 (537.33)		2163.02 (557.27)		2146.94 (605.93)		2098.67 (483.58)	
**Physical activity (IPAQ-L^c^; min/week)**									
	Intervention	921.92 (768.43)		1131.86 (906.70)		1127.57 (977.58)		1033.20 (824.76)	
	Control	966.16 (1012.76)		851.95 (828.80)		900.97 (733.54)		878.42 (614.80)	

^a^Intervention (n=78) and control (n=75) groups over 4 measurement time points.

^b^The time points were baseline (t0), after the 12-week intervention (t1), and after an additional 6 months (t2) and 12 months (t3) of follow-up.

^c^IPAQ-L: long version of the International Physical Activity Questionnaire.

Reported total physical activity (IPAQ-L) increased significantly in the intervention group in the short term (t0 to t1; *P*=.03), with a slightly significant difference between the groups (*P*=.049). No significant changes were observed in physical activity measured by the activity tracker.

### Cardiometabolic Variables

The descriptive results of cardiometabolic variables are shown in Table 4, and the corresponding effect sizes are presented in Multimedia Appendix 4. No group differences in cardiometabolic variables were noted during the intervention (Multimedia Appendix 5). Only HDL cholesterol increased significantly at t3 compared with t0 in the intervention group (*P*=.046).

**Table 4:** Descriptive statistics of cardiometabolic variables (intention-to-treat analysis).

Variable and group^a^			t0^b^, mean (SD)		t1^b^, mean (SD)		t2^b^, mean (SD)		t3^b^, mean (SD)
**Fasting glucose (mg/dL)**									
	Intervention	88.88 (7.67)		88.37 (8.64)		87.79 (7.95)		87.39 (10.26)	
	Control	89.60 (12.07)		90.00 (9.25)		89.49 (9.39)		88.32 (11.77)	
**HbA_1c_^c^ (%)**									
	Intervention	5.44 (0.33)		5.42 (0.32)		5.46 (0.25)		5.47 (0.28)	
	Control	5.37 (0.52)		5.37 (0.36)		5.43 (0.32)		5.45 (0.29)	
**Total cholesterol (mg/dL)**									
	Intervention	211.79 (34.39)		207.60 (33.06)		206.31 (33.89)		207.11 (33.54)	
	Control	218.79 (48.51)		211.07 (45.19)		213.19 (38.11)		211.03 (40.82)	
**LDL^d^ cholesterol (mg/dL)**									
	Intervention	132.62 (29.76)		129.35 (28.03)		141.79 (29.28)		140.44 (27.47)	
	Control	137.48 (40.14)		133.57 (35.11)		146.71 (30.65)		143.99 (32.96)	
**HDL^e^ cholesterol (mg/dL)**									
	Intervention	57.62 (11.16)		57.78 (9.42)		60.45 (9.17)		60.98 (11.73)	
	Control	57.59 (11.92)		56.48 (10.79)		59.31 (10.34)		58.09 (9.20)	
**Triglycerides (mg/dL)**									
	Intervention	108.04 (47.29)		105.60 (42.05)		103.78 (45.32)		101.55 (44.76)	
	Control	121.73 (65.47)		116.67 (67.98)		115.36 (55.59)		114.10 (53.04)	
**Systolic blood pressure (mmHg)**									
	Intervention	128.98 (13.43)		126.88 (13.47)		129.22 (11.62)		126.50 (11.17)	
	Control	130.20 (14.09)		126.36 (13.74)		132.12 (16.83)		128.27 (13.17)	
**Diastolic blood pressure (mmHg)**									
	Intervention	87.99 (9.08)		86.16 (8.70)		87.86 (7.68)		86.23 (7.12)	
	Control	87.11 (8.47)		84.82 (8.71)		88.78 (9.78)		86.79 (8.44)	

^a^Intervention (n=78) and control (n=75) groups over 4 measurement time points.

^b^The time points were baseline (t0), after the 12-week intervention (t1), and after an additional 6 months (t2) and 12 months (t3) of follow-up.

^c^HbA_1c_: glycated hemoglobin.

^d^LDL: low-density lipoprotein.

^e^HDL: high-density lipoprotein.

## Discussion

The main finding of this study was that the interactive web-based health program focusing on dietary energy density showed positive effects on body weight, fat mass, and waist circumference. These effects were significantly more pronounced in the intervention group than in the control group with only web-based knowledge transfer and were also evident in the longer-term 12-month follow-up period. With regard to short-term weight loss from web-based interventions (3-4 months), comparable interventions reported short-term weight loss of 2.0 kg [42], 2.3 kg [43], and 4.2 kg [44], while the participants in the intervention group in this study lost 3.98 kg in the ITT analysis. At the 12-month follow-up, the weight loss of participants in the intervention group in this study was 4.18 kg in the ITT analysis, which is above the reported weight loss for comparable interventions of 0.9 kg [45] and 2.1 kg [46] after 12 months. In a recent meta-analysis, weight loss for web-based interventions ranged from 1.3 to 6.2 kg [25]. With 3.98 to 4.18 kg weight loss in the intervention group, depending on the time of measurement, the weight loss for the interactive web-based intervention in this study is in the upper middle range compared with the findings for other web-based interventions. Web-based interventions with expert contact achieved better results in some cases [47], but have limited comparability with a fully automated web-based intervention owing to the potentially limited range and higher costs.

Fat mass in the intervention group decreased significantly compared with the finding in the control group over the course of the study, whereas fat-free mass reduced in the intervention group, but did not differ between the 2 groups. Both of these are important findings because few web-based interventions perform body composition measurements and most use self-reported parameters. This limits the accuracy of the measurements and does not provide direct information regarding the loss of body fat. Moreover, it is known that weight loss interventions can result in a loss of fat-free mass and muscle mass [48]. Preservation of fat-free mass during weight loss is an important goal because, in addition to high fat mass [49], low fat-free mass is associated with high mortality [50] and plays an important role in energy expenditure [51]. Besides the amount of fat mass, the fat distribution is of great importance. Thus, visceral obesity has long been identified as a risk factor for type 2 diabetes or higher all-cause mortality [52,53]. In this regard, the interactive web-based weight loss program was able to show an above-average reduction in waist circumference compared with the findings for other web-based interventions [54].

The effectiveness of the interactive web-based health program was also partially evident in behavioral variables, such as energy density, energy intake, fat intake, and self-reported physical activity. Based on the focus of the program, the reduction in energy density in the short and long term in the intervention group was an important finding. Energy intake developed consistently with energy density in the intervention group, although the effect was significant only in the short term. The increase in energy density at t2 in both groups could be due to a seasonal effect [55], as the measurement time point t2 was from October 2020 to February 2021 for the majority of participants. However, the COVID-19 pandemic restrictions may also have had effects on energy density. For example, it has already been shown that a COVID-19 lockdown can lead to increased intake of foods with high energy density [56]. Aside from changes in the diet, a short-term increase in total physical activity reported using IPAQ-L was also observed in the intervention group. This increase in total activity was not reflected in the activity tracker data. The possible causes could be measurement inaccuracies of the activity tracker and an overestimation of the participants in the intervention group.

Despite the positive effects on body weight and composition, the improvements in cardiometabolic variables in the intervention group were small and not significantly superior to the findings in the control group. For some variables, such as systolic and diastolic blood pressure, improvements were similar to those with other web-based interventions, whereas LDL cholesterol showed a worse trend [28]. One possible explanation for the small changes in cardiometabolic variables may be insufficient weight loss. A 5% weight loss is considered necessary to achieve a clinically relevant effect [57]. Less than half (33/78, 42.3%) of the participants in the intervention group achieved more than 5% weight loss at t3. Although this proportion is substantially greater compared with the finding in a recent study by Beleigoli et al [58], it may be insufficient for changes in cardiometabolic variables in this study population. Another reason could be that the cardiometabolic variables in the study population were, on average, close to or within the normal ranges. Despite being overweight or obese, the study participants were predominantly metabolically healthy, and therefore, the potential for improvements in cardiometabolic variables was low. Both possible explanations are supported by the analysis of Morris et al [59], who studied changes in cardiometabolic risk factors per kg of body weight loss. Thus, in a metabolically healthy study population, the expected effects on individual cardiometabolic risk factors are small at about 4 kg weight loss.

The results of this study confirm the benefits of lowering energy density shown by several previous studies in relation to weight loss [12,13], but indicate, with respect to the control group, that communicating the energy density concept alone is not sufficient. Rather, it seems to require appropriate feedback in terms of energy density, as was the case in the interactive web-based intervention. Therefore, an interactive web-based intervention as the medium and the energy density concept as the content could be an appropriate combination.

Several limitations must be considered in this study. First, complete blinding was not possible due to the recognition of the program by the participants. Therefore, the motivation of the participants might have been influenced based on the recognition of the program. Second, both study groups might have been additionally motivated to attain their health goals by interest in free medical examinations as well as activity trackers received as incentives. Conversely, the participants of the clinical substudy might have been more motivated than the participants of the online study already at the beginning, since participation in medical examinations required more commitment. Third, the effect of the COVID-19 pandemic on both groups was difficult to quantify. Qualitative feedback from study participants indicated that the COVID-19 pandemic and its associated restrictions affected people positively, negatively, or not at all with regard to their weight management, dietary, or physical activity behaviors. Descriptive examinations of physical activity during the first lockdown in March 2020 showed, on average, negligible change in measured physical activity. Fourth, nonuse might have affected the effectiveness of the program and the study results. Overall, however, the usage analysis showed that the program was used multiple times by all participants in the intervention group of this study (data not shown). Because the control group received only web-based information without updates, one-time use was expected. However, despite these limitations, this study provides evidence that an interactive and fully automated web-based health program focusing on energy density concepts exerts positive effects on weight loss among people with overweight and obesity. The standardized measurement of body weight and clinical variables in this study, compared with the self-reported measurement of body weight in other studies, is a strength of this study. Owing to the potentially unlimited availability of fully automated web-based programs, there may be relevant public health effects.

This clinical study showed that an interactive web-based weight loss program was effective and superior to a noninteractive web-based weight loss program. The interactivity of web-based interventions seems to play an important role in effectiveness. Based on the present results, pure knowledge transfer is not sufficient to induce sustainable weight loss and adherence to a diet with low energy density. In the web-based intervention studied here, interactivity was established via feedback on dietary documentation and activities achieved or not achieved. Future research should consider in more detail what and how much interactivity is necessary to make web-based weight loss interventions effective. Nevertheless, our results suggest that integrating the concept of a diet with low energy density into an interactive web-based weight loss program is useful for achieving weight loss.

## Appendix

Multimedia Appendix 1 Effect sizes of anthropometric variables (intention-to-treat analysis).

Multimedia Appendix 2 Results of the robust linear mixed model of behavioral variables (intention-to-treat analysis).

Multimedia Appendix 3 Effect sizes of behavior variables (intention-to-treat analysis).

Multimedia Appendix 4 Effect sizes of cardiometabolic variables (intention-to-treat analysis).

Multimedia Appendix 5 Results of the robust linear mixed model of cardiometabolic variables (intention-to-treat analysis).

Multimedia Appendix 6 CONSORT EHEALTH Checklist (V 1.6.1).

## Abbreviations

HDL: high-density lipoprotein
IPAQ-L: long version of the International Physical Activity Questionnaire
ITT: intention to treat
LDL: low-density lipoprotein
PP: per protocol
SEVERA: Section for Health Services Research and Rehabilitation Research

## Data Availability

The data sets generated during or analyzed during this study are available from the corresponding author on reasonable request.
