# Supplementary material for: Effects of a Web-Based Lifestyle Intervention on Weight Loss and Cardiometabolic Risk Factors in Adults With Overweight and Obesity: Randomized Controlled Clinical Trial
Source: J Med Internet Res. 2023 Jun 27;25:e43426. doi: 10.2196/43426 (PMC10337343; doi:10.2196/43426)
Supplement: Multimedia Appendix 1 [file jmir_v25i1e43426_app1.docx]

**Multimedia Appendix 1.** Effect sizes of anthropometric variables (intention-to-treat analysis).^a,b^

| **Group** | **t0-t1** | **t0-t2** | **t0-t3** |
| --- | --- | --- | --- |
| **Body weight** | | | |
| Intervention | -0.39 [-0.71, -0.08] | -0.44 [-0.76, -0.12] | -0.42 [-0.73, -0.01] |
| Control | -0.12 [-0,44, 0.20] | -0.09 [-0.41, 0.23] | -0.11 [-0.43, 0.21] |
| **Fat mass** | | | |
| Intervention | -0.47 [-0.79, -0.15] | -0.36 [-0.67, -0.04] | -0.45 [-0.77, -0.14] |
| Control | -0.20 [-0.52, 0.12] | -0.05 [-0.37, 0.27] | -0.10 [-0.42, 0.23] |
| **Fat-free mass** | | | |
| Intervention | -0.14 [-0.45, 0.18] | -0.21 [-0.52, 0.11] | -0.21 [-0.53, 0.11] |
| Control | -0,04 [-0.36, 0.28] | -0.14 [-0,46, 0.18] | -0.06[-0.38, 0.26] |
| **Waist circumference** | | | |
| Intervention | -0.37 [-0.68, -0.05] | -0.44 [-0.76, -0.12] | -0.55 [-0.87, -0.23] |
| Control | -0.16 [-0,48, 0.17] | -0.21 [-0.54, 0.11] | -0.18 [-0.50, 0.14] |

^a^Cohen *d* with 95% CI.

^b^Interpretation: |d| = 0.2: small effect, |d| = 0.5: medium effect, |d| = 0.8: large effect.
