# Supplementary material for: Effects of a Web-Based Lifestyle Intervention on Weight Loss and Cardiometabolic Risk Factors in Adults With Overweight and Obesity: Randomized Controlled Clinical Trial
Source: J Med Internet Res. 2023 Jun 27;25:e43426. doi: 10.2196/43426 (PMC10337343; doi:10.2196/43426)
Supplement: Multimedia Appendix 2 [file jmir_v25i1e43426_app2.docx]

**Multimedia Appendix 2.** Results of the robust linear mixed model of behavioral variables (intention-to-treat analysis).

| Predictor | | Energy density (kcal/g) | | Energy intake (kcal/day) | | Protein intake (g/day) | | Carbohydrate intake (g/day) | | Fat intake (g/day) | | Alcohol intake (g/day) | | Fiber intake (g/day) | | Physical activity (Fitbit; (min/week) | | Physical activity (IPAQ-L^a^; min/week) | | |
| --- | --- | --- | --- | --- | --- | --- | --- | --- | --- | --- | --- | --- | --- | --- | --- | --- | --- | --- | --- | --- |
|  | | Estimate^b^ | *P* value | Estimate^b^ | *P* value | Estimate^b^ | *P* value | Estimate^b^ | *P* value | Estimate^b^ | *P* value | Estimate^b^ | *P* value | Estimate^b^ | *P* value | Estimate^b^ | *P* value | Estimate^b^ | *P* value |  |
|  | |  |  |  |  |  |  |  |  |  |  |  |  |  |  |  |  |  |  |  |
| Intercept | | 1.58 (0.08) | <.001 | 1910.12 (134.80) | <.001 | 67.00 (5.30) | <.001 | 198.79 (15.65) | <.001 | 79.03 (6.37) | <.001 | 7.06 (1.81) | <.001 | 21.41 (1.94) | <.001 | 2242.15 (144.07) | <.001 | 765.31 (143.07) | <.001 |  |
| **Time^c^** | |  |  |  |  |  |  |  |  |  |  |  |  |  |  |  |  |  |  |  |
|  | t0-t1 | −0.38 (0.10) | <.001 | −334.76 (133.39) | .01 | −1.72 (5.60) | .76 | −21.14 (16.99) | .21 | −24.18 (6.91) | <.001 | −2.19 (1.99) | .27 | 1.55 (2.07) | .45 | 46.15 (151.26) | .76 | 358.42 (158.96) | .03 |  |
|  | t0-t2 | −0.15 (0.10) | .15 | 18.07 (131.59) | .89 | 2.50 (5.44) | .65 | 4.53 (17.98) | .80 | −8.30 (7.63) | .28 | −2.87 (1.98) | .15 | 2.70 (2.09) | .20 | 14.18 (189.36) | .94 | 253.15 (169.56) | .14 |  |
|  | t0-t3 | −0.27 (0.10) | .007 | −257.78 (144.43) | .08 | 1.49 (5.92) | .80 | −20.96 (16.98) | .22 | −17.15 (7.29) | .02 | −1.92 (2.11) | .36 | 2.32 (2.46) | .35 | −37.39 (158.06) | .81 | 236.24 (171.28) | .17 |  |
| Group (control) | | 0.03 (0.05) | .58 | 31.20 (85.67) | .72 | 4.12 (3.40) | .22 | 4.91 (9.96) | .62 | 0.26 (4.05) | .95 | −0.32 (1.15) | .78 | −0.85 (1.23) | .49 | −20.68 (91.86) | .82 | −10.74 (90.76) | .91 |  |
| **Time×group (control)** | |  |  |  |  |  |  |  |  |  |  |  |  |  |  |  |  |  |  |  |
|  | t0-t1 | 0.15 (0.07) | .02 | 113.50 (85.20) | .18 | −0.38 (3.64) | .92 | 1.12 (10.80) | .92 | 10.71 (4.43) | .02 | 1.09 (1.23) | .38 | −1.15 (1.31) | .38 | −38.94 (96.47) | .69 | −200.18 (101.77) | .049 |  |
|  | t0-t2 | 0.10 (0.07) | .15 | −71.71 (83.59) | .39 | −2.60 (3.56) | .46 | −9.76 (11.01) | .38 | 2.18 (4.85) | .65 | 0.98 (1.29) | .45 | −1.12 (1.31) | .39 | −23.79 (114.27) | .84 | −138.42 (105.27) | .19 |  |
|  | t0-t3 | 0.13 (0.06) | .04 | 86.86 (96.36) | .37 | −1.86 (3.67) | .61 | 3.16 (11.04) | .775 | 7.21 (5.43) | .11 | 1.11 (1.31) | .40 | −1.14 (1.54) | .46 | −21.75 (99.63) | .83 | −109.95 (108.59) | .31 |  |

^a^IPAQ-L: long version of the International Physical Activity Questionnaire.

^b^Unstandardized regression coefficients with standard errors in parentheses.

^c^The time points were baseline (t0), after the 12-week intervention (t1), and after an additional 6 months (t2) and 12 months (t3) of follow-up.
