# Supplementary material for: Effects of a Web-Based Lifestyle Intervention on Weight Loss and Cardiometabolic Risk Factors in Adults With Overweight and Obesity: Randomized Controlled Clinical Trial
Source: J Med Internet Res. 2023 Jun 27;25:e43426. doi: 10.2196/43426 (PMC10337343; doi:10.2196/43426)
Supplement: Multimedia Appendix 3 [file jmir_v25i1e43426_app3.docx]

**Multimedia Appendix 3.** Effect sizes of behavior variables (intention-to-treat analysis).^a,b^

| **Group** | **t0-t1** | **t0-t2** | **t0-t3** |
| --- | --- | --- | --- |
| **Energy density** | | | |
| Intervention | -0.72 [-1.05, -0.40] | -0.23 [-0.54, 0.09] | -0.44 [-0.76, -0.12] |
| Control | -0.20 [-0.52, 0.12] | 0.10 [-0.22, 0.42] | -0.03 [-0.35, 0.30] |
| **Energy intake** | | | |
| Intervention | -0.36 [-0.67, -0.04] | -0.08 [-0.40, 0.23] | -0.23 [-0.54, 0.09] |
| Control | -0.22 [-0.55, 0.10] | -0.23 [-0.56, 0.09] | -0.17 [-0.49, 0.15] |
| **Protein intake** | | | |
| Intervention | -0.09 [-0.41, 0.22] | -0.01 [-0.32, 0.31] | 0.00 [-0.31, 0.32] |
| Control | -0.18 [-0.50, 0.14] | -0.22 [-0.54, 0.10] | -0.18 [0.50, 0.14] |
| **Carbohydrate intake** | | | |
| Intervention | -0.29 [-0.60, 0.03] | -0.12 [-0.43, 0.20] | -0.24 [-0.56,0.08] |
| Control | -0.21 [-0.53, 0.11] | -0.15 [-0.48, 0.17] | -0.17 [-0.49, 0.15] |
| **Fat intake** | | | |
| Intervention | -0.48 [-0.80, -0.16] | -0.12 [-0.44, 0.19] | -0.27 [-0.58, 0.05] |
| Control | -0.15 [-0.47, 0.18] | -0.21 [-0.54, 0.11] | -0.14 [-0.46, 0.19] |
| **Alcohol intake** | | | |
| Intervention | -0.13 [-0.44, 0.18] | -0.28 [-0.60, 0.04] | -0.13 [-0.45, 0.18] |
| Control | -0.11 [-0.43, 0.21] | -0.14 [-0.46, 0.18] | -0.02 [-0.34, 0.30] |
| **Fiber intake** | | | |
| Intervention | 0.02 [-0.30, 0.33] | 0.16 [-0.15, 0.48] | 0.16 [-0.15, 0.48] |
| Control | -0.15 [-0.47, 0.17] | -0.03 [-0.35, 0.30] | -0.07 [-0.39, 0.25] |
| **Physical activity (Fitbit)** | | | |
| Intervention | -0.12 [-0.44, 0.19] | -0.07 [-0.39, 0.24] | -0.18 [-0.49, 0.37] |
| Control | -0.05 [-0.37, 0.27] | -0.08 [-0.40, 0.24] | -0.18 [-0.50, 0.14] |
| **Physical activity (IPAQ-L)** | | | |
| Intervention | 0.25 [-0.07, 0.57] | 0.23 [-0.08, 0.55] | 0.14 [-0.18, 0.45] |
| Control | -0.12 [-0.44, 0.20] | -0.07 [-0.39, 0.25] | -0.11 [-0.43, 0.22] |

^a^Cohen *d* with 95% CI.

^b^Interpretation: |d| = 0.2: small effect, |d| = 0.5: medium effect, |d| = 0.8: large effect
