# Supplementary material for: Effects of a Web-Based Lifestyle Intervention on Weight Loss and Cardiometabolic Risk Factors in Adults With Overweight and Obesity: Randomized Controlled Clinical Trial
Source: J Med Internet Res. 2023 Jun 27;25:e43426. doi: 10.2196/43426 (PMC10337343; doi:10.2196/43426)
Supplement: Multimedia Appendix 4 [file jmir_v25i1e43426_app4.docx]

**Multimedia Appendix 4.** Effect sizes of cardiometabolic variables (intention-to-treat analysis).^a,b^

| **Group** | **t0-t1** | **t0-t2** | **t0-t3** |
| --- | --- | --- | --- |
| **Fasting glucose** | | | |
| Intervention | -0.06 [-0.38, 0.25] | -0.14 [-0.45, 0.18] | -0.17 [-0.48, 0.15] |
| Control | 0.04 [-0.28, 0.36] | -0.01 [-0.33, 0.31] | -0.11 [-0.43, 0.21] |
| **HbA_1c_** | | | |
| Intervention | -0.07 [-0.38, 0.04] | 0.04 [-0.28, 0.35] | 0.08 [-0.24, 0.39] |
| Control | 0.01 [-0.31, 0.33] | 0.15 [-0.18, 0.47] | 0.19 [-0.13, 0.51] |
| **Total cholesterol** | | | |
| Intervention | -0.12 [-0.44, 0.19] | -0.16 [-0.48, 0.15] | -0.13 [-0.45, 0.18] |
| Control | -0.17 [-0.49, 0.16] | -0.13 [-0.45, 0.19] | -0.17 [-0.49, 0.15] |
| **LDL cholesterol** | | | |
| Intervention | -0.11 [-0.43, 0.20] | 0.31 [-0.01, 0.63] | 0.27 [-0.04, 0.59] |
| Control | -0.10 [-0.42, 0.12] | 0.26 [-0.06, 0.58] | 0.18 [-0.14, 0.50] |
| **HDL cholesterol** | | | |
| Intervention | 0.02 [-0.30, 0.33] | 0.28 [-0.04, 0.59] | 0.29 [-0.02, 0.61] |
| Control | -0.10 [-0,42, 0.22] | 0.15 [-0.17, 0.47] | 0.05 [-0.27, 0.37] |
| **Triglycerides** | | | |
| Intervention | -0.06 [-0.37, 0.26] | -0.09 [-0.41, 0.22] | -0.14 [-0.46, 0.17] |
| Control | -0.08 [-0.40, 0.25] | -0.11 [-0.43, 0.22] | -0.13 [-0.45, 0.19] |
| **Systolic blood pressure** | | | |
| Intervention | -0.16 [-0.47, 0.16] | 0.02 [-0.30, 0.33] | -0.20 [-0.52, 0.11] |
| Control | -0.28 [-0.60, 0.05] | 0.12 [-0.20, 0.44] | -0.14 [-0.46, -0.18] |
| **Diastolic blood pressure** | | | |
| Intervention | -0.21 [-0.52, 0.11] | -0.02 [-0.33, 0.30] | -0.22 [-0.53, 0.01] |
| Control | -0.27 [-0.59, 0.06] | 0.18 [-0.14, 0.50] | -0.04 [-0.36, 0.28] |

^a^Cohen *d* with 95% CI.

^b^Interpretation: |d| = 0.2: small effect, |d| = 0.5: medium effect, |d| = 0.8: large effect.
