# Supplementary material for: Effects of a Web-Based Lifestyle Intervention on Weight Loss and Cardiometabolic Risk Factors in Adults With Overweight and Obesity: Randomized Controlled Clinical Trial
Source: J Med Internet Res. 2023 Jun 27;25:e43426. doi: 10.2196/43426 (PMC10337343; doi:10.2196/43426)
Supplement: Multimedia Appendix 5 [file jmir_v25i1e43426_app5.docx]

**Multimedia Appendix 5.** Results of the robust linear mixed model of cardiometabolic variables (intention-to-treat analysis).

| Predictor | | Fasting glucose (mg/dL) | | HbA_1c_^a^ (%) | | Total cholesterol (mg/dL) | | LDL^b^ cholesterol (mg/dL) | | HDL^c^ cholesterol (mg/dL) | | Triglycerides (mg/dL) | | Systolic blood pressure (mmHg) | | Diastolic blood pressure (mmHg) | |
| --- | --- | --- | --- | --- | --- | --- | --- | --- | --- | --- | --- | --- | --- | --- | --- | --- | --- |
|  | | Estimate^d^ | *P* value | Estimate^d^ | *P* value | Estimate^d^ | *P* value | Estimate^d^ | *P* value | Estimate^d^ | *P* value | Estimate^d^ | *P* value | Estimate^d^ | *P* value | Estimate^d^ | *P* value |
|  | |  |  |  |  |  |  |  |  |  |  |  |  |  |  |  |  |
| Intercept | | 89.51 (2.06) | <.001 | 5.52 (0.07) | <.001 | 207.15 (10.35) | <.001 | 128.65 (8.18) | <.001 | 57.16 (2.66) | <.001 | 93.43 (10.66) | <.001 | 126.31 (3.29) | <.001 | 87.97 (2.23) | <.001 |
| **Time^e^** | |  |  |  |  |  |  |  |  |  |  |  |  |  |  |  |  |
|  | t0-t1 | −2.55 (2.10) | .23 | −0.09 (0.06) | .12 | −5.28 (8.00) | .51 | −3.11 (6.42) | .62 | 1.01 (2.22) | .65 | 4.43 (9.38) | .37 | −0.18 (3.18) | .95 | −0.98 (2.06) | .63 |
|  | t0-t2 | −2.95 (2.38) | .22 | −0.03 (0.06) | .58 | −9.97 (8.77) | .26 | 5.55 (6.57) | .40 | 3.20 (2.21) | .14 | 8.00 (9.30) | .39 | −1.43 (3.37) | .67 | −1.23 (2.52) | .63 |
|  | t0-t3 | −2.62 (2.20) | .24 | −0.01 (0.06) | .84 | −3.19 (7.88) | .69 | 8.59 (7.01) | .23 | 4.45 (2.22) | .046 | −6.12 (9.17) | .51 | −2.98 (3.35) | .37 | −2.42 (2.33) | .30 |
| Group (control) | | −0.45 (1.31) | .73 | −0.08 (0.04) | .07 | −0.39 (5.12) | .49 | 3.43 (5.20) | .51 | 0.05 (1.70) | .97 | 7.68 (6.82) | .26 | 1.66 (2.09) | .43 | −0.42 (1.42) | .77 |
| **Time×group (control)** | |  |  |  |  |  |  |  |  |  |  |  |  |  |  |  |  |
|  | t0-t1 | 1.66 (1.35) | .22 | 0.05 (0.04) | .15 | −0.39 (5.12) | .94 | −0.53 (4.11) | .90 | −1.09 (1.40) | .44 | −4.37 (6.00) | .47 | −1.99 (2.03) | .33 | −0.83 (1.32) | .53 |
|  | t0-t2 | 1.63 (1.49) | .28 | 0.05 (0.04) | .16 | 2.69 (5.55) | .63 | 3.04 (4.24) | .47 | −0.46 (1.37) | .74 | 1.85 (6.01) | .76 | 1.36 (2.20) | .54 | 1.37 (1.59) | .39 |
|  | t0-t3 | 0.62 (1.40) | .66 | 0.05 (0.04) | .26 | −1.66 (5.06) | .74 | −0.74 (4.55) | .87 | −1.97 (1.37) | .15 | 0.13 (5.95) | >.99 | 0.29 (2.11) | .89 | 0.88 (1.44) | .54 |

^a^HbA_1c_: glycated hemoglobin.

^b^LDL: low-density lipoprotein.

^c^HDL: high-density lipoprotein.

^d^Unstandardized regression coefficients with standard errors in parentheses.

^e^The time points were baseline (t0), after the 12-week intervention (t1), and after an additional 6 months (t2) and 12 months (t3) of follow-up.
